# Endothelial IGF-1/IGF-1R signaling supports young-blood-induced restoration of blood–brain barrier integrity and microvascular architecture in aged mice: evidence from the heterochronic parabiosis model

**DOI:** 10.1007/s11357-026-02424-3

**Published:** 2026-08-20

**Authors:** Rafal Gulej, Rebeka Kristof, Roland Patai, Siva Sai Chandragiri, Shoba Ekambaram, Mark Nagykaldi, Evelyn Brunner, Peter Mukli, Adam Nyul-Toth, Dorina Nagy, Kiana Vali Kordestan, Raghavendra Yelahanka Nagaraja, Santny Shanmugarama, Andriy Yabluchanskiy, Stefano Tarantini, Zoltan Ungvari, Anna Csiszar

**Affiliations:** 1 Vascular Cognitive Impairment, Healthy Brain Aging Program, Department of Neurosurgery, University of Oklahoma Health Sciences Center Neurodegeneration, Oklahoma City, OK USA; 2 Oklahoma Center for Geroscience and Healthy Brain Aging, University of Oklahoma Health Sciences Center, Oklahoma City, OK USA; 3 International Training Program in Geroscience, Doctoral College, Health Sciences Division/Institute of Preventive Medicine and Public Health, Semmelweis University, Budapest, Hungary; 4 Fodor Center for Prevention and Healthy Aging, Semmelweis University, Budapest, Hungary; 5 International Training Program in Geroscience, Doctoral College/Institute of Clinical Pathophysiology, Semmelweis University, Budapest, Hungary; 6 Institute for Translational Research, Budapest, Hungary; 7 Stephenson Cancer Center, University of Oklahoma, Oklahoma City, OK USA

**Keywords:** Aging, Heterochronic parabiosis, Cell non-autonomous, Neuroendocrine, Blood–brain barrier, Cerebral microcirculation, Endothelial function, Neuroinflammation, Insulin-like growth factor 1 (IGF-1), IGF-1 receptor (IGF-1R), Endothelial IGF-1R deficiency, Systemic IGF-1 knockdown, Vascular cognitive impairment and dementia, VCID, Cerebrovascular aging, Rejuvenation, Neurovascular unit

## Abstract

Aging is associated with blood–brain barrier (BBB) breakdown and microvascular rarefaction, key contributors to cerebral neuroinflammation, hypoperfusion, and vascular cognitive impairment and dementia (VCID). Exposure to a young systemic milieu through heterochronic parabiosis has been shown to restore BBB integrity and enhance cerebrovascular function in aged mice, suggesting that circulating factors can rejuvenate the aging brain. However, the molecular mediators responsible for these effects remain poorly defined. Circulating insulin-like growth factor-1 (IGF-1) declines markedly with age and has been implicated in endothelial dysfunction and BBB disruption, raising the possibility that IGF-1/IGF-1 receptor (IGF-1R) signaling contributes to the vascular rejuvenation induced by young blood. To test this hypothesis, we combined heterochronic parabiosis with complementary transgenic approaches targeting either endothelial IGF-1R deletion (VE-Cadherin-CreERT2/Igf1rfl/fl) or systemic IGF-1 knockdown (TBG-Cre-AAV8/Igf1fl/fl). BBB permeability to fluorescent tracers and cortical microvascular density were quantified by intravital two-photon microscopy through chronic cranial windows. We found that exposure of aged mice to young circulation markedly reduced BBB leakage and increased cortical capillary density. These beneficial effects were significantly attenuated in aged parabionts lacking endothelial IGF-1R or paired with young partners deficient in systemic IGF-1, demonstrating that both endothelial receptor activation and circulating IGF-1 availability are required for full cerebrovascular rejuvenation. Despite this attenuation, partial improvement persisted, indicating that IGF-1/IGF-1R signaling contributes to, but does not fully account for, the vascular benefits of young blood. These findings identify the IGF-1/IGF-1R axis as an important mediator of young blood–induced restoration of BBB integrity and microvascular density. Targeting downstream effectors of IGF-1R signaling, together with lifestyle interventions that enhance somatotropic axis responsiveness, may offer geroscience-guided strategies to preserve BBB function and cognitive resilience in aging.

## Introduction

Aging leads to progressive deterioration of the cerebral microcirculation, marked by capillary rarefaction, endothelial dysfunction, impaired regulation of cerebral blood flow, and breakdown of the blood–brain barrier (BBB) [1–4] the specialized interface that safeguards the homeostatic environment of the central nervous system. These microvascular alterations play a pivotal role in age-related cognitive decline and contribute to the pathogenesis of vascular cognitive impairment and dementia (VCID) [1, 3, 5]. BBB dysfunction allows entry of plasma proteins, inflammatory mediators, and neurotoxic molecules into the brain parenchyma, activating microglia, promoting neuroinflammation, and impairing neuronal function [1–3]. The resulting inflammatory and oxidative milieu drives aberrant synaptic pruning, contributes to demyelination, and accelerates white matter damage, the hallmarks of cerebral small vessel disease and VCID [1–3]. Leakage of fibrinogen and other plasma constituents further promotes microglial activation and complement-mediated synaptic loss, while endothelial and perivascular inflammation disrupt axonal integrity and oligodendrocyte homeostasis [1–3]. Together, these processes establish a self-perpetuating cycle of BBB breakdown, neuroinflammation, and neurodegeneration that progressively compromises brain connectivity and cognitive performance with age [1–3]. In parallel, microvascular rarefaction diminishes cerebral perfusion and oxygen delivery, further exacerbating neuronal vulnerability [6–9]. Together, these processes constitute the microvascular contributions to age-related cognitive impairment and are also thought to play a significant role in the pathogenesis of Alzheimer’s disease (AD) [1–3].

Although many hallmarks of cerebrovascular aging, such as oxidative stress, mitochondrial dysfunction, and cellular senescence, are driven by cell-intrinsic mechanisms, accumulating evidence indicates that systemic, cell-non-autonomous influences strongly modulate vascular aging [10, 11]. Circulating factors derived from endocrine organs, liver, adipose tissue, and immune cells dynamically regulate endothelial phenotype and BBB permeability [12]. The heterochronic parabiosis model, in which the circulations of young and aged animals are surgically joined [13–19], has revealed that exposure to a youthful systemic environment reverses multiple features of vascular aging, including endothelial dysfunction, microvascular rarefaction, and neurovascular uncoupling [12, 20]. Conversely, exposure of young animals to aged circulation rapidly induces vascular aging phenotypes, underscoring the bidirectional influence of the circulating milieu on cerebromicrovascular health [12, 21]. Our previous parabiosis studies demonstrated that aged mice exposed to young circulation exhibit restored BBB integrity and increased cortical capillary density [8]. These findings established that anti-geronic circulating factors in young blood regulate barrier function and angiogenic processes in the cerebral microcirculation. However, the molecular mediators of these rejuvenating effects remained unidentified.

Among the numerous circulating factors implicated in heterochronic parabiosis, IGF-1 was selected for mechanistic investigation because substantial prior evidence linked age-related reductions in IGF-1 signaling to cerebrovascular dysfunction, while independent transcriptomic studies identified activation of IGF-1R-dependent pathways as a prominent feature of vascular rejuvenation in heterochronic parabionts. These observations provided a strong rationale for directly testing the contribution of IGF-1/IGF-1R signaling to young blood-mediated cerebrovascular rejuvenation. Independent lines of research from our group and others have established that the insulin-like growth factor-1 (IGF-1)/IGF-1 receptor (IGF-1R) axis plays a key role in maintaining microvascular integrity and BBB function throughout the lifespan [22–24]. Growth hormone (GH)–dependent hepatic IGF-1 production declines markedly with age (the “somatopause” [25, 26]), leading to systemic IGF-1 deficiency [27, 28] and reduced IGF-1R input to endothelial cells. IGF-1 supports endothelial nitric oxide synthase activation, mitochondrial function, angiogenesis, and tight-junction maintenance, while limiting oxidative stress and apoptosis [22, 24, 29, 30]. Consequently, reduced circulating IGF-1 or endothelial IGF-1R expression contributes to endothelial senescence, microvascular rarefaction, and BBB leakage [23, 24, 31, 32]. Recent transcriptomic profiling of vascular tissues from heterochronic parabionts revealed reactivation of IGF-1R-dependent pathways as a consistent signature of vascular rejuvenation [33]. Moreover, our complementary work on neurovascular coupling demonstrated that the recovery of functional hyperemic responses in aged parabionts depends, in part, on both circulating IGF-1 and endothelial IGF-1R signaling [34]. These converging observations led us to hypothesize that IGF-1/IGF-1R signaling also mediates, at least in part, the rejuvenating effects of young blood on BBB integrity and microvascular architecture.

To test this hypothesis, we combined heterochronic parabiosis with complementary transgenic approaches targeting either systemic IGF-1 production (TBG-Cre-AAV8/Igf1^fl/fl^) or endothelial IGF-1R expression (VE-Cadherin-Cre^ERT2^/Igf1r^fl/fl^). Using high-resolution intravital two-photon microscopy through chronic cranial windows, we quantified BBB permeability to multiple fluorescent tracers of different molecular weights and measured cortical microvascular density and branching complexity. This dual-transgenic, cross-circulation approach allowed us to dissect the relative contributions of endocrine and endothelial IGF-1 signaling to the restoration of BBB integrity and microvascular density in aged mice exposed to young blood, and to determine whether this pathway represents a key, though not exclusive, mediator of systemic cerebrovascular rejuvenation.

## Materials and methods

### Transgenic mouse models and animal husbandry

**Fig. 1:**
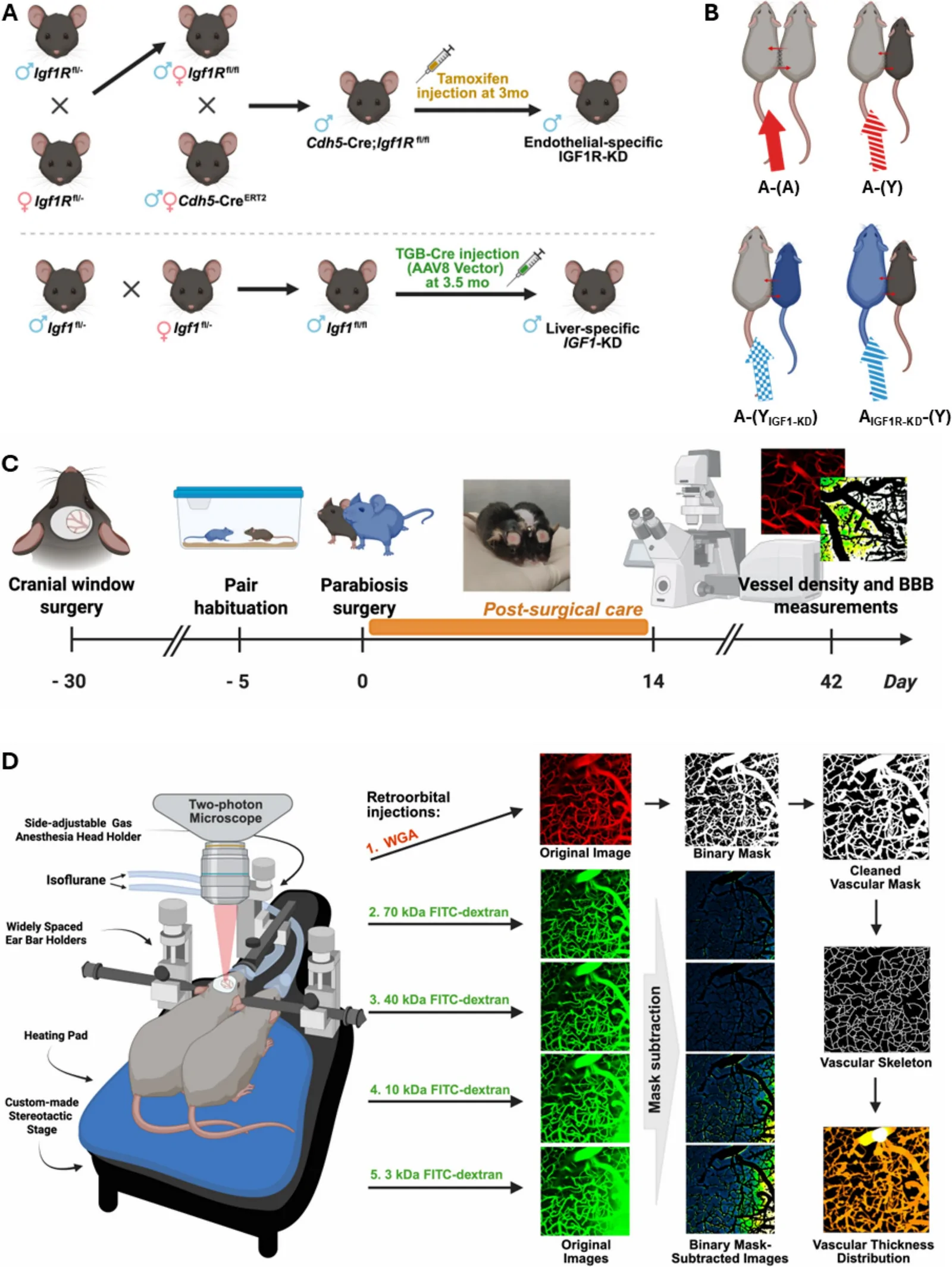
Experimental design. **A** Breeding strategy used to generate tamoxifen-inducible endothelial-specific IGF1R knockdown mice (*Cdh5*-Cre^ERT2^;*Igf1r*^fl/fl^) and mice with decreased circulating IGF-1 levels (*Igf1*^fl/fl^), generated by liver-specific delivery of TGB-Cre using the AAV8 system. **B** Schematic representation of the four experimental parabiosis groups used in this study. **C)** Experimental timeline showing the timing of cranial window surgery, pair habituation, parabiosis surgery, post-surgical care, and the subsequent assessment of the BBB integrity and microvascular density using intravital two-photon microscopy. **D** Illustration of parabionts positioned under the two-photon microscope, with an overview of image acquisition, processing, and analysis. Mo, month-old; KD, knock down; BBB, blood–brain barrier; WGA, wheat germ agglutinin; kDa, kilodalton

To generate a model with inducible endothelial IGF-1 receptor deficiency, mice carrying floxed Igf1r alleles (B6;129-Igf1r^tm2Arge^/J, Jackson Laboratory) were crossed with VE-Cadherin-Cre^ERT2^ mice (C57BL/6-Tg(Cdh5-CreERT2)1Rha; Taconic Biosciences) [23]. At 3 months of age, offspring received intraperitoneal tamoxifen injections (75 mg/kg/day, dissolved in corn oil) for five consecutive days to activate Cre recombinase and delete *Igf1r* selectively in endothelial cells. Control littermates were injected with corn oil alone (Fig. 1A).

Systemic suppression of IGF-1 production was achieved using male homozygous Igf1^fl/fl^ mice (B6.129(FVB)-Igf1^tm1Dlr^/J; loxP sites flanking exon 4; Jackson Laboratory, backcrossed to the C57BL/6 background [29, 35–38]). In these animals, Cre-mediated excision produces a truncated, biologically inactive form of IGF-1. Hepatocyte-specific deletion was induced through intravenous administration of adeno-associated virus serotype 8 (AAV8) vectors expressing Cre recombinase under the thyroxine-binding globulin (TBG) promoter (AAV8.TBG.PI.Cre.rBG, AddGene, USA) [29, 35–38] or control vectors expressing eGFP (AAV8.TBG.PI.eGFP.WPRE.bGH, AddGene, USA). At 3.5 months of age, mice were anesthetized with isoflurane and injected retro-orbitally with 1.3 × 10^10^ viral genomes in 100 µL of sterile saline. The TBG promoter restricts Cre activity to hepatocytes, resulting in a liver-specific decline in circulating IGF-1 (Fig. 1A).

All animals were housed in the Rodent Barrier Facility of the University of Oklahoma Health Campus (OUHC) under specific pathogen-free conditions, maintained on a 12-h light/dark cycle, and provided ad libitum access to a standard AIN-93G diet and water. Wild-type C57BL/6 mice from the NIA colony (Charles River Laboratories) were used as parabiotic partners. All experiments were performed preferentially using male mice. Male animals were selected to minimize variability associated with sex-specific differences in endocrine aging trajectories, circulating IGF-1 levels, and cerebrovascular responses.

Parabiosis was performed using 4.5-month-old (young) and 18-month-old (aged) mice, resulting in four experimental groups: (1) aged endothelial IGF-1R–deficient parabionts paired with young wild-type partners (AIGF1R-KD-(Y)); (2) aged wild-type parabionts paired with IGF-1–deficient young partners (A-(YIGF1-KD)); (3) wild-type heterochronic aged parabionts (A-(Y)); and (4) wild-type isochronic aged parabionts (A-(A)). Parentheses denote the non-measured partner (Fig. 1B). Wild-type parabionts were not generated in the present study; instead, control data for young reference parabionts were obtained from a parallel cohort examined under identical experimental and analytical conditions [8].

Following surgery, animals were returned to fresh cages and received comprehensive postoperative care. Daily health monitoring was conducted by research staff and OUHSC veterinary personnel.

All procedures, including housing, surgeries, imaging, and euthanasia, were performed in accordance with protocols approved by the OUHC Institutional Animal Care and Use Committee (protocols #21–031 and #24-021).

### Parabiosis surgery

Parabiosis was established between the following pairs: young and aged wild-type mice; two aged wild-type mice; aged VE-Cadherin-Cre^ERT2^/Igf1r^fl/fl^ and young wild-type mice; and aged wild-type and young TBG-Cre/Igf1^fl/fl^ mice, following previously described methods [8, 39].

To facilitate social adaptation, candidate partners were co-housed for 5 days before surgery (Fig. 1C). On the day of the procedure, animals were anesthetized with isoflurane (3% for induction and 1–2.5% for maintenance in medical air, 0.6–0.8 L/min flow rate) and positioned on sterile drapes under separate vaporizers to permit individualized anesthesia control. The lateral flanks were shaved and disinfected alternately with povidone–iodine and 70% ethanol. Longitudinal skin incisions were made from the knee to the neck, opposing scapulae and femora were gently exposed using sterile scalpel and sutured together using 4–0 silk sutures. Finally, the skin edges were joined using continuous pattern of 4–0 silk sutures to establish stable vascular anastomosis.

Perioperative care included subcutaneous administration of buprenorphine, meloxicam, warmed saline, and enrofloxacin to ensure adequate analgesia, hydration, and antimicrobial prophylaxis. Supportive pharmacotherapy and monitoring were continued for 1 week postoperatively. Thereafter, animals were monitored twice daily for wound healing, hydration, body weight, and general well-being during the first 2 weeks. On postoperative day 14, skin sutures were removed under isoflurane anesthesia (3% for induction and 1–2% for maintenance in medical air at a flow rate of 0.6–0.8 L/min). Following suture removal, parabionts were monitored daily until the experimental endpoint.

### Intravital two-photon microscopy and quantification of BBB permeability

To assess BBB integrity and cortical microvascular structure, we performed longitudinal intravital two-photon imaging through chronic cranial windows, as previously validated for quantifying vascular permeability and capillary density [7, 8].

### Cranial window preparation

At the age of 17 months, wild type and endothelial-specific IGF1-R-deficient male mice were implanted with glass cranial windows 1 month before the parabiosis surgery, as described previously [8]. Briefly, mice were anesthetized with isoflurane (3% for induction and 1–2% for maintenance in medical air at a flow rate of 0.6–0.8 L/min) and positioned in a stereotaxic frame, with the head securely fixed using ear bars and a nose mask (51625, Stoelting, USA). Scalp hair was removed, and the skin was disinfected with sterile saline followed by alternating applications of 70% ethanol and povidone–iodine. Local anesthesia was achieved by topical application of a 2% lidocaine solution in saline. The scalp was then excised, and the periosteum was gently retracted to expose the skull. A circular craniotomy (~ 4 mm in diameter) was performed over the right somatosensory cortex using a microdrill, taking care to preserve the dura mater. The exposed cortical surface was irrigated with sterile saline and immediately sealed with a 5-mm glass coverslip (64–0700, Warner Instruments, USA), affixed with cyanoacrylate glue and further secured with acrylic cement (51,459, Stoelting, USA). During surgery, mice received subcutaneous injections of buprenorphine (1 mg/kg; Buprenorphine ER, 1 mg/mL; ZooPharm, USA) and enrofloxacin (10 mg/kg; 2.27%; Baytril, Elanco, USA) for analgesia and antimicrobial prophylaxis. Animals were continuously monitored until full recovery and received daily antibiotic treatment and close clinical monitoring for 3 days postoperatively. Chronic cranial window implantation has been extensively validated for longitudinal studies of cerebrovascular structure and blood–brain barrier permeability [7]. To minimize procedure-related inflammation and barrier disruption, imaging was performed following a 1-month recovery period, which exceeds the interval previously shown to permit restoration of baseline BBB integrity and cerebrovascular physiology [7].

### Imaging procedures

Blood–brain barrier permeability and cortical microvascular density were assessed in vivo using two-photon microscopy, as described previously [8]. Cranial window-equipped parabionts were anesthetized using isoflurane and positioned in a stereotactic frame to stabilize the head and minimize motion artifacts. Imaging was performed using a FluoView 1000 MPE two-photon microscope (Olympus, Japan) coupled to a Ti:Sapphire laser tuned to 800 nm for FITC–dextran excitation (MaiTai HP DeepSee-OL, Spectra-Physics, USA) and equipped with an XLPLN25XWMP 25 × water-immersion objective (1.05 numerical aperture; Olympus, Japan).

To label the microvasculature and identify an optimal imaging region, parabionts received a retro-orbital injection of wheat germ agglutinin (WGA-Alexa Fluor 594, 1 mg/mL; 4 µL/g body weight; W11262, Thermo Fisher Scientific, USA). A Z-stack of cortical images (30 optical sections, 5 µm intervals; 150 µm total depth) was then acquired starting at the cortical surface (baseline).

Subsequently, FITC-conjugated dextran tracers of decreasing molecular weights (70 kDa, 40 kDa, 10 kDa, and 3 kDa; 4 µL/g body weight, 2 mg/mL; D1823, D1845, D1821, and D3305; Thermo Fisher Scientific, USA) were injected retro-orbitally in a sequence. Following each injection, a 15-min time-lapse series (hyperstack) was recorded, consisting of 15 Z-stacks (30 images each, 5 µm step size) captured at one-minute intervals (extravasation imaging). All images were acquired under identical detector gain settings for consistency across animals. Laser power was adjusted individually to optimize visualization of parenchymal vasculature and FITC–dextran extravasation and was kept constant between baseline and subsequent imaging sessions. Throughout imaging, body temperature was maintained at 37 ± 0.5 °C using a feedback-controlled heating pad, and oxygen saturation and heart rate were monitored by pulse oximetry (MouseOx, Starr Life Sciences).

### Image analysis

All imaging data were processed using ImageJ (version 1.53t, NIH, USA) following our previously established image processing and analysis protocol [8]. Briefly, Z-stacks from baseline and post-tracer imaging were combined and corrected for three-dimensional drift and motion artifacts using the *Correct 3D Drift* plugin. The uppermost meningeal vessels (first 50 µm from the cortical surface) were excluded from analysis to focus on parenchymal microvasculature within a depth range of 50–150 µm. Vascular masks were generated using the Weka Segmentation tool applied to the maximal intensity projection of the first WGA-labeled time frame. These binary vascular masks were subsequently used for the quantification of tracer extravasation, microvascular density, branching complexity, and vessel size distribution (Fig. 1D).

Blood–brain barrier permeability was quantified as the cumulative increase in extravascular FITC fluorescence intensity relative to baseline (*I/I*_*0*_) for each tracer and averaged for each experimental group.

For microvascular morphology analysis, autofluorescent puncta were removed from two-dimensional vascular images to prevent false-positive segmentation. To further minimize artifacts, objects smaller than 25 pixels [2] were excluded from the binarized masks, and vessel edges were refined using the *Dilate* and *Erode* functions. The cleaned vascular masks were then skeletonized, and the following parameters were extracted and normalized to the imaging area: (1) skeleton lengths for the assessment of the vessel density, (2) number of branches for the Microvascular Branching Index (MBI), and (3) number of junctions for the Microvascular Complexity Index (MCI).

Finally, vessel size distribution was quantified using the *LocalThickness* plugin, which color-codes structures based on their local width and generates histograms of vessel diameters. Vessels ≤ 10 µm in diameter were defined as capillaries, accounting for a slight overestimation due to image segmentation and smoothing (Fig. 1D).

### Statistical analysis

All statistical analyses and graph preparation were performed using GraphPad Prism (version 10.1.0; GraphPad Software, San Diego, CA, USA). Data were analyzed using one-way ANOVA followed by Sidak’s post hoc test. Outliers were identified using Grubbs’ test (*α* = 0.05); one data point was excluded on this basis. Each experimental group included 5–9 biological replicates, with exact N stated in the figure legend. Results are presented as mean ± standard deviation (SD). Statistical significance was accepted at *p* < 0.05, p values for each comparison are provided within the graphs.

## Results

### Young blood restores blood–brain barrier integrity in aged parabionts through IGF-1/IGF-1R–dependent mechanisms

We first examined whether the rejuvenating effect of young circulation on the blood–brain barrier depends on intact IGF-1/IGF-1R signaling. Four parabiosis groups were studied (Fig. 1A): (1) wild-type isochronic aged pairs (A–(A)), (2) wild-type heterochronic aged parabionts (A–(Y)), (3) aged endothelial IGF-1R–deficient parabionts paired with young wild-type partners (A_IGF1R-KD_–(Y)), and (4) aged wild-type parabionts paired with young IGF-1–deficient partners (A–(Y_IGF1-KD_)).

**Fig. 2:**
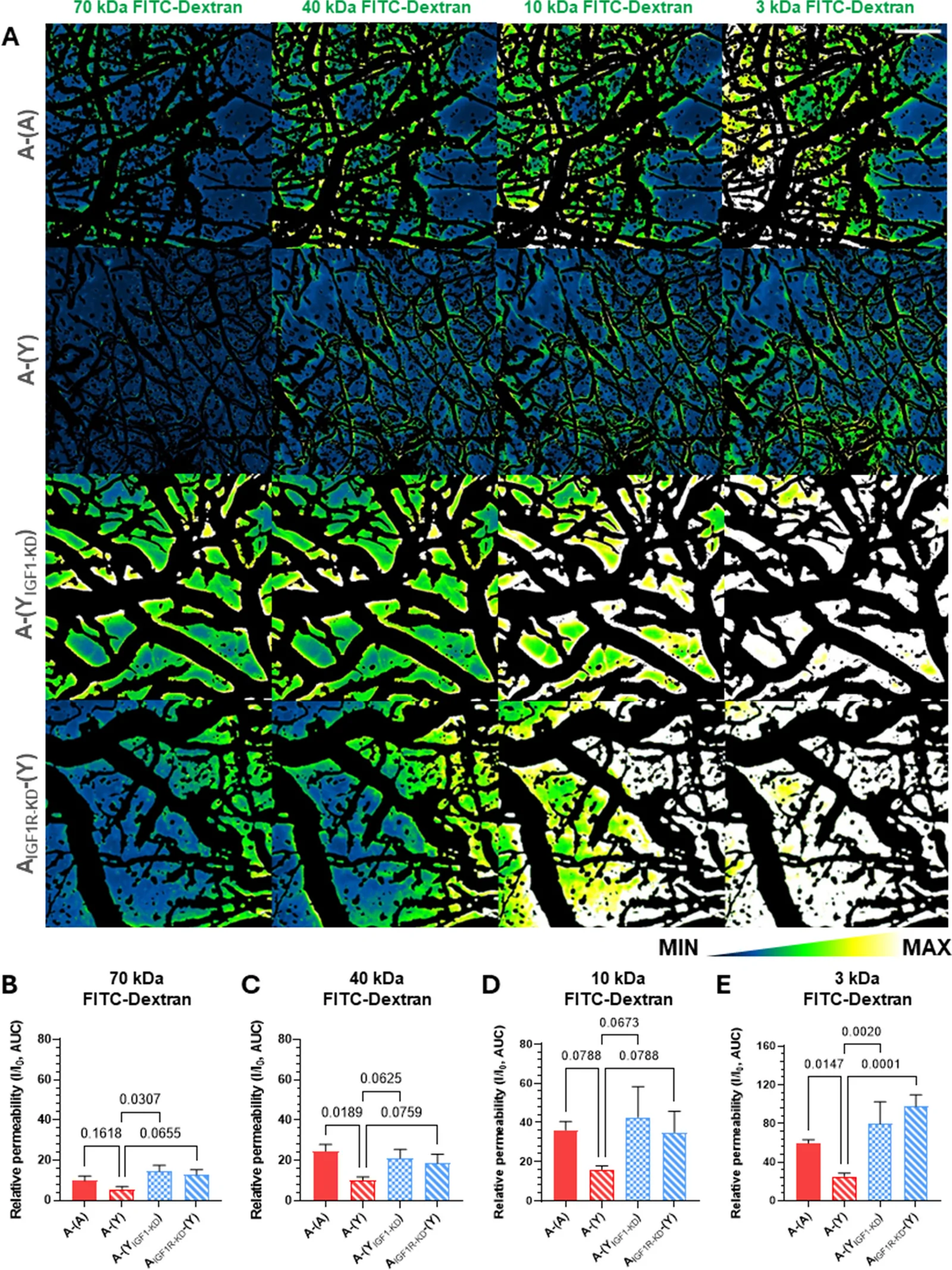
IGF-1/IGF-1R signaling contributes to young blood–mediated rejuvenation of blood–brain barrier integrity in heterochronic aged parabionts. **A** Representative intravital two-photon microscopy images illustrating blood–brain barrier (BBB) permeability in each experimental group. Pseudocolor images depict extravasation of intravenously injected FITC-conjugated dextrans of decreasing molecular weight (70 kDa, 40 kDa, 10 kDa, and 3 kDa). The heat bar indicates the color scale for tracer extravasation, with dark blue representing minimal leakage and yellow-to-white representing maximal extravasation. Scale bar, 100 µm. **B**–**E** Quantification of tracer extravasation, expressed as cumulative fluorescence intensity (normalized to baseline) within the extravascular space for each fluorescent tracer in individual parabionts. Data are presented as mean ± SD; *n* = 7, 8, 6, and 5 parabionts per group. Statistical analysis was performed using one-way ANOVA with Holm–Šidák post hoc correction. Data for FITC–dextran extravasation in wild-type isochronic (A–(A)) and heterochronic (A–(Y)) parabionts were previously published by our laboratory [8]

BBB permeability was assessed by intravital two-photon microscopy using fluorescent dextran tracers of different molecular weights (3, 10, 40, and 70 kDa). Representative images and quantitative analyses revealed extensive parenchymal tracer extravasation in aged isochronic parabionts, consistent with pronounced BBB disruption (Fig. 2A). In contrast, aged parabionts exposed to a young milieu exhibited significantly reduced extravasation across all tracer sizes, confirming BBB rejuvenation by young blood (Fig. 2A).

However, this protective effect was attenuated when IGF-1/IGF1-1R signaling was disrupted. Aged parabionts lacking endothelial IGF-1R (A_IGF1R-KD_–(Y) or paired with young partners with hepatocyte-specific IGF-1 deficiency (A–(Y_IGF1-KD_) showed increased tracer extravasation compared with wild-type heterochronic aged parabionts (Fig. 2A). Quantitative analysis demonstrated that BBB permeability to the largest used tracer (70 kDa FITC-dextran) was significantly elevated in aged parabionts paired with IGF-1–deficient young partners (*p* = 0.0307) and showed a strong trend toward elevation in endothelial IGF-1R–deficient aged parabionts (*p* = 0.0655) relative to wild-type heterochronic controls (Fig. 2B–E). Similarly, BBB permeability to 40 and 10 kDa FITC–dextrans remained elevated in both IGF-1– and IGF-1R–deficient conditions compared with wild-type heterochronic pairs (A_IGF1R-KD_–(Y): *p* = 0.0759 and 0.0788; A–(Y_IGF1-KD_): *p* = 0.0625 and 0.0673, respectively). Notably, permeability to the smallest tracer (3 kDa FITC–dextran) was significantly increased in both conditions (p = 0.0001 for A_IGF1R-KD_–(Y); *p* = 0.002 for A–(Y_IGF1-KD_)), with mean values exceeding those observed in isochronic aged parabionts. Collectively, these results indicate that both circulating IGF-1 and endothelial IGF-1R signaling are required for the full BBB-restoring effect of young blood.

### Systemic IGF-1 signaling promotes cortical microvascular regeneration in aged mice exposed to young circulation

**Fig. 3:**
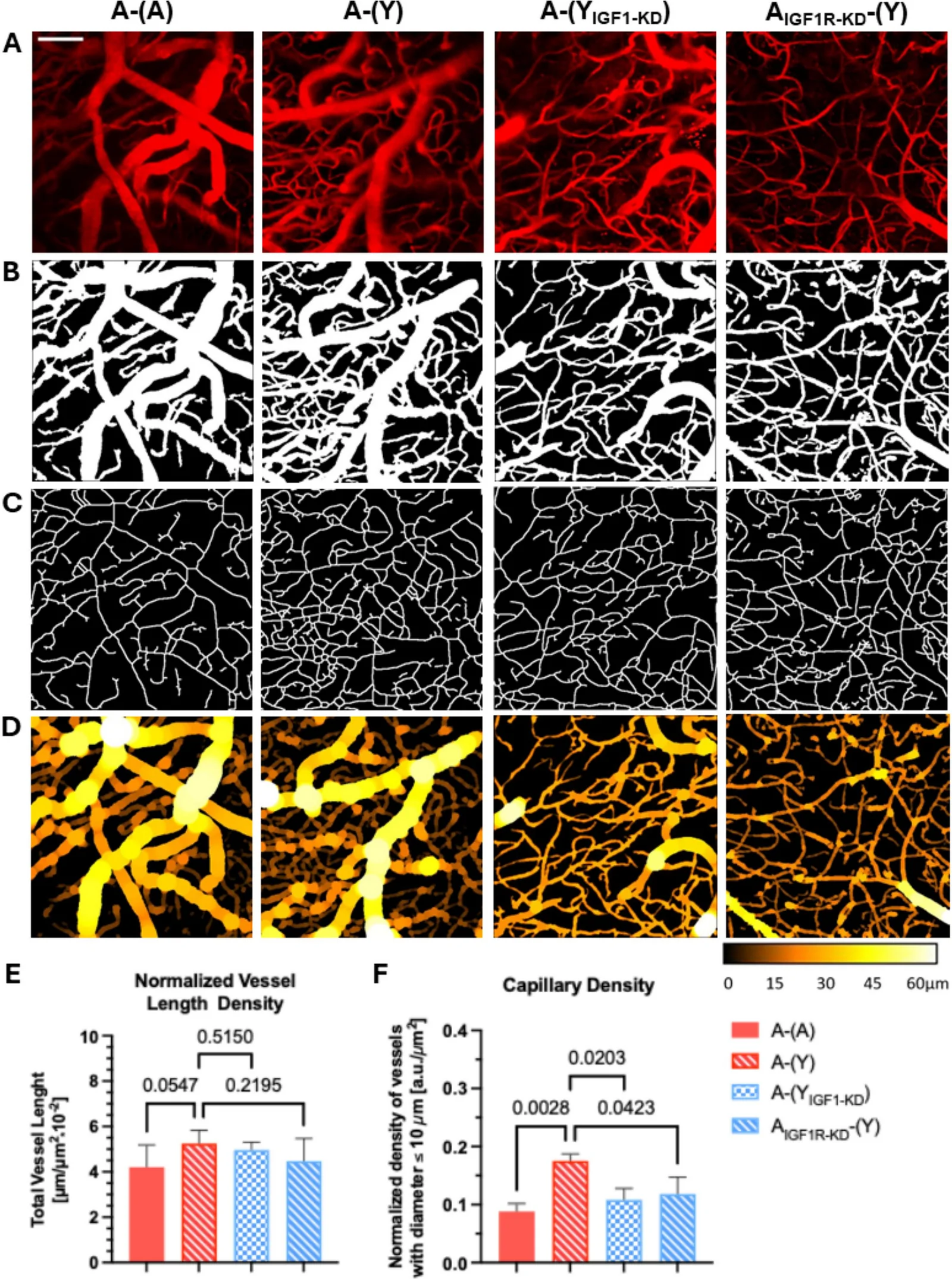
Young-blood-mediated improvements in microvascular density, complexity, and capillarization are diminished in IGF-1/IGF-1R-deficient heterochronic aged parabionts. **A** Representative intravital two-photon microscopy images of the cerebromicrovasculature in the somatosensory cortex (imaging depth: 50–150 µm) from each experimental group. Cerebral vessels were labeled with wheat germ agglutinin. Scale bar, 100 µm. **B** Original two-photon images were segmented to create a binary mask of the vasculature. **C, D** Binary masks were further processed to generate vascular skeletons (**C**) and vessel diameter distribution maps (**D**). Skeletonized images of cerebrovasculature were used to quantify vessel length density (**E**) for each experimental group. Vessel diameter distribution maps were used to generate histograms of the vessel size distribution in each experimental groups. Area under the curve for the vessels with diameters ≤ 10 µm was quantified for each parabiont. **F** Normalized density of vessels with diameters ≤ 10 µm for each experimental group. Data are presented as mean ± SD; *N* = 8, 8, 7, and 5 parabionts per group. Statistical analysis was performed using one-way ANOVA with Holm–Šidák post hoc correction; *p* < 0.05. Data for wild-type isochronic (A–(A)) and heterochronic (A–(Y)) aged parabionts were previously published in Gulej et al., Geroscience (2024) [8]

We next examined whether IGF-1/IGF-1R signaling also contributes to microvascular remodeling in aged parabionts. Three-dimensional reconstructions of cortical capillary networks revealed pronounced capillary rarefaction and reduced branching complexity in aged isochronic parabionts (A-(A)), consistent with established features of vascular aging. In contrast, exposure to a young milieu (A–(Y)) markedly increased total microvascular and capillary density, (Fig. 3E, F), indicating robust structural rejuvenation of the cortical microvasculature.

Disruption of IGF-1/IGF-1R signaling attenuated these pro-angiogenic effects of young blood. In aged endothelial IGF-1R–deficient parabionts paired with young wild-type partners (A_IGF1R-KD_–(Y)) and in aged wild-type parabionts paired with young IGF-1–deficient partners (A–(Y_IGF1-KD_), the pro-angiogenic effect of young blood was partially diminished. Specifically, microvascular density was lower than that observed in wild-type heterochronic aged parabionts, although these differences did not reach statistical significance (*p* = 0.2195 and 0.515, respectively; Fig. 3E). Similarly, microvascular branching and complexity indices were lower when compared with wild-type heterochronic aged parabionts, although these differences were also not statistically significant (*p* > 0.05, data not shown). Notably, all three vascular parameters remained higher than those measured in aged isochronic parabionts, suggesting partial preservation of young blood–mediated microvascular remodeling despite impaired IGF-1 signaling. Although these findings were directionally consistent with a partial loss of young blood-induced vascular rejuvenation, the study was not powered to definitively detect modest differences in these structural endpoints. Accordingly, these observations should be interpreted as suggestive trends that warrant confirmation in larger cohorts.

In contrast to these microvascular metrics, capillary-specific analysis revealed a critical dependence on intact IGF-1/IGF-1R signaling. Quantification of capillary density, defined as the normalized density of vessels with ≤ 10 µm diameter, demonstrated a significant reduction in both aged endothelial IGF-1R–deficient parabionts and aged parabionts paired with IGF-1–deficient young partners (*p* = 0.0423 and *p* = 0.0203, respectively; Fig. 3F). These findings suggest that both endocrine IGF-1 availability and endothelial IGF-1R responsiveness are essential for optimal microvascular regeneration, yet additional circulating or local mechanisms are likely to contribute to the overall rejuvenation process.

## Discussion

Our findings demonstrate that the rejuvenating effects of a young systemic milieu on the aging cerebral microvasculature critically depend on endothelial IGF-1/IGF-1R signaling. Specifically, heterochronic parabiosis restored blood–brain barrier integrity [8] and increased cortical microvascular density [8] in aged wild-type mice, whereas these benefits were markedly attenuated in aged parabionts lacking endothelial IGF-1R or exposed to young circulation deficient in systemic IGF-1. These results identify the IGF-1/IGF-1R axis as a mechanistic mediator of the cerebrovascular rejuvenation conferred by young blood. Importantly, the rejuvenating effects of young blood were attenuated rather than completely abolished by endothelial IGF-1R deficiency or systemic IGF-1 depletion. This observation indicates that IGF-1/IGF-1R signaling is a critical contributor to cerebrovascular rejuvenation but is unlikely to represent the sole mediator of the phenomenon. Rather, our findings support a model in which multiple circulating anti-geronic factors act in parallel or synergistically to restore cerebrovascular health during heterochronic parabiosis. The residual protective effects observed in the present study underscore the complexity of the rejuvenating systemic milieu [11, 12] and suggest that complementary pathways remain active even when IGF-1 signaling is impaired.

Endothelial IGF-1R signaling exerts multiple protective effects on the BBB [22, 38, 40, 41]. Previous work has shown that activation of this pathway maintains tight-junction integrity [22, 23, 41, 42] and mitochondrial function [59–62], counteracting matrix-degrading and inflammatory processes that destabilize the barrier. Our current findings extend this paradigm by demonstrating that the absence of endothelial IGF-1R negates the beneficial impact of young circulation on BBB permeability. This suggests that IGF-1R activation is necessary for the reestablishment of barrier selectivity in aged brains exposed to young blood. The BBB, however, is not an isolated endothelial structure but an integral component of the neurovascular unit (NVU), where astrocytes, pericytes, and endothelial cells cooperate to regulate barrier permeability and cerebrovascular homeostasis. Importantly, our previous studies demonstrated that astrocytes also express functional IGF-1 receptors, and that astrocyte-specific IGF-1R knockdown impairs neurovascular function in young mice, phenocopying the vascular aging phenotype [43]. These observations suggest that IGF-1 signaling within both endothelial and astrocytic compartments may serve as an important effector mechanism through which circulating IGF-1 preserves NVU integrity and barrier function. In aging, diminished IGF-1 bioavailability [27, 28] weakens this multicellular communication network and may contribute to the progressive breakdown of the BBB and the consequent accumulation of neurotoxic plasma proteins, leukocyte infiltration, and neuroinflammation, contributing to the pathogenesis of VCID^1–3,25^. Restoration of BBB integrity may also have important consequences extending beyond barrier permeability itself. By limiting neuroinflammation, reducing exposure of neural tissue to circulating neurotoxic factors, and preserving the homeostatic environment of the neurovascular unit, improved BBB function is expected to support neurovascular coupling, cerebral perfusion, and ultimately cognitive resilience [1, 3]. Indeed, our recent studies demonstrated that exposure to young blood restores neurovascular coupling responses in aged parabionts, suggesting that multiple components of neurovascular function are responsive to systemic rejuvenation [34, 39]. In addition to endothelial cells, astrocytes, pericytes, and inflammatory cells likely contribute to the coordinated restoration of neurovascular unit function observed during heterochronic parabiosis. Future studies integrating behavioral testing, neurovascular functional assessments, and longitudinal imaging will be important to determine whether the observed improvements in BBB integrity and microvascular architecture translate into meaningful cognitive benefits during aging.

In parallel with BBB restoration, young blood exposure enhanced cortical capillarization in aged parabionts [8], consistent with prior reports linking youthful systemic factors to angiogenic reactivation [12]. This structural rejuvenation was lost when endothelial IGF-1R was deleted or systemic IGF-1 was suppressed, implicating this pathway in the maintenance of microvascular density [31, 36]. IGF-1R signaling promotes endothelial proliferation, migration, and survival [44–47] via downstream activation of eNOS and mitochondrial biogenesis pathways; therefore, its reactivation through young blood likely facilitates angiogenesis and prevents age-related microvascular rarefaction. These effects complement the previously observed protective effects on neurovascular coupling mechanisms [29], collectively suggesting that IGF-1–dependent endothelial plasticity contributes to the functional and structural rejuvenation of the aging cerebral microvasculature. It is important to note that the present study quantified structural microvascular endpoints and was not designed to directly assess angiogenic activity. Therefore, the observed increase in capillary density should be interpreted as evidence of microvascular remodeling and restoration of vascular architecture rather than direct proof of ongoing angiogenesis. While these findings are consistent with enhanced vascular plasticity in response to a youthful systemic environment, future studies incorporating endothelial proliferation markers and assessments of vessel remodeling dynamics will be required to define the precise cellular mechanisms underlying these structural adaptations.

While our results highlight a pivotal role for IGF-1/IGF-1R signaling, they also indicate that young blood-mediated rejuvenation is multifactorial [12]. Factors including other endocrine regulators and NAD⁺-related metabolites have been implicated in overlapping vascular protective pathways [12]. It is conceivable that IGF-1R signaling interacts synergistically with these factors, amplifying endothelial resilience and promoting mitochondrial rejuvenation. Conversely, pro-geronic components of aged plasma, including GDF-15 and TNFα, may suppress IGF-1R signaling or its downstream effectors [12]. Thus, the balance between anti- and pro-geronic signals in the circulation likely dictates the net outcome on cerebromicrovascular aging [12]. The present findings also provide a framework for future mechanistic investigations. IGF-1R activation regulates multiple downstream pathways relevant to BBB maintenance and vascular remodeling, including endothelial nitric oxide synthase activation, mitochondrial homeostasis, tight-junction protein expression, inflammatory signaling and microvascular autoregulatory function [31, 38, 40, 43, 48–53]. Future transcriptomic and proteomic studies may help define the molecular programs activated downstream of IGF-1R and identify additional circulating factors that cooperate with IGF-1 to mediate cerebrovascular rejuvenation.

Microvascular rarefaction, neurovascular dysfunction, and BBB disruption are recognized as major contributors to cerebral hypoperfusion, white matter damage, and cognitive decline in aging [1–3, 5, 54–57]. By elucidating a mechanistic link between systemic IGF-1 decline and cerebromicrovascular dysfunction, our findings bridge endocrine aging and neurovascular pathology [24]. Although direct systemic IGF-1 replacement is unlikely to be a feasible therapeutic option due to oncogenic and metabolic risks, strategies that enhance endogenous IGF-1 signaling capacity may offer safer and more physiologic approaches to preserve cerebromicrovascular health [31, 58]. Regular physical exercise, healthy diets, and metabolic interventions that optimize growth hormone–IGF-1 axis responsiveness could help maintain endothelial IGF-1 sensitivity and downstream protective signaling. Targeting these adaptive mechanisms, potentially in combination with pharmacological interventions that act synergistically with downstream effectors of IGF-1R signaling, may represent a viable path to sustain BBB integrity, microvascular density, and cognitive resilience in aging.

Several limitations of the present study should be acknowledged. First, while heterochronic parabiosis is a powerful tool to study systemic regulation of aging, it introduces potential confounders such as surgical stress, altered metabolism, and shared organ systems that may independently influence vascular outcomes. Second, endothelial IGF-1R deficiency induced by VE-cadherin–driven Cre recombination is not limited to the brain vasculature and may affect peripheral endothelial beds, indirectly modifying circulating or metabolic factors that influence the BBB. Third, systemic IGF-1 knockdown in hepatocytes may alter multiple downstream cell-non-autonomous pathways, complicating strict attribution of effects to IGF-1 alone. Fourth, the study was not designed to identify specific circulating factors beyond IGF-1 that act in concert to restore BBB integrity and vascular density; these mechanisms will require future omics-based and interventional approaches. Finally, while we demonstrate structural and permeability improvements, whether these translate into preserved or improved cognitive outcomes remains to be tested.

In summary, heterochronic parabiosis experiments using genetic models of endothelial IGF-1R deficiency and systemic IGF-1 knockdown demonstrate that the IGF-1/IGF-1R signaling axis is an important contributor to young blood–induced rejuvenation of the BBB and cortical microvascular network. Future work should focus on defining how IGF-1R signaling interfaces with other rejuvenating pathways, such as NAD⁺/SIRT1 signaling, at the cellular and molecular levels [12]. Integrating cell-type-resolved transcriptomic and proteomic profiling with dynamic in vivo imaging of barrier function will be critical to map the hierarchy and cross-talk of these networks and to determine their impact on neurocognitive outcomes. Such systems-level insight may ultimately guide the development of geroscience-based interventions that preserve vascular integrity and cognitive resilience during aging.

## Data Availability

N/A.
